# Storage stability of hospital-prepared mianserin suppositories: evaluation of residual active ingredient content and bacteriological contamination under different storage conditions

**DOI:** 10.1186/s40780-026-00543-9

**Published:** 2026-02-07

**Authors:** Rei Tanaka, Tomoe Ichikawa, Junya Sato

**Affiliations:** 1 Department of Pharmacotherapy, Faculty of Pharmaceutical Sciences, Shonan University of Medical Sciences, Kanagawa, Japan; 2 Department of Microbiology and Immunology, Faculty of Pharmaceutical Sciences, Shonan University of Medical Sciences, Kanagawa, Japan

**Keywords:** Mianserin, Suppositories, Hospital preparations, Stability, Pharmaceutical storage

## Abstract

**Background:**

Mianserin suppositories are a hospital preparation usable for patients who cannot take oral medication. However, data on the stability of hospital preparations are limited. Therefore, this study investigated the stability of mianserin suppositories over time and the appropriate storage methods.

**Methods:**

For the long-term chemical stability testing, five conditions were compared: RL; room temperature (15–30°C) under ambient light, RD; room temperature in the dark, CL; cool temperature (2–8°C) under ambient light, CD; cool temperature in the dark, and FD; frozen temperature (−20°C) in the dark. High-performance liquid chromatography (HPLC) was used to compare time-dependent component contents at 0, 2, 4, 8, 16, and 24 weeks (*n* = 5). Microbial limit tests for total aerobic microorganisms and fungi were conducted using the agar plate dilution method immediately after preparation and after 24 weeks in RL, and after 24 weeks at FD (*n* = 4).

**Results:**

A gradual decline was observed under all storage conditions. The residual content rate (mean ± standard error (SE)) after 24 weeks was as follows: RL, 96.2 ± 0.3%; RD, 96.2 ± 0.2%; CL, 97.0 ± 0.3%; CD, 97.3 ± 0.3%; and FD, 98.3 ± 0.2%. Under all conditions—immediately after preparation, after 24 weeks of RL, and after 24 weeks of FD—the total aerobic microbial count was ≤1000 colony-forming units (CFU)/g, and the total fungal count was ≤100 CFU/g.

**Conclusion:**

The results showed that the residual content remained satisfactory above 95% from immediately after preparation through 24 weeks under all storage conditions, ranging from RL to FD.

## Introduction

Mianserin suppositories are hospital preparations made by crushing approved mianserin tablets and mixing them with the base agent, witepsol (H-15). Hospital preparations are defined as having “formulations prepared by hospital pharmacists at each medical institution under the Medical Care Act for patients with diseases or conditions that cannot be adequately treated with routinely supplied pharmaceuticals.” This is considered one of the foundational elements of personalized medicine tailored to each individual [1]. On the other hand, the use of hospital preparation constitutes off-label use and is therefore not covered by the Relief Services for Adverse Health Effects on PMDA.

In recent years, an increasing number of patients have experienced difficulty taking oral medications due to age-related decline in swallowing function [2] and the growing number of cancer survivors following gastrointestinal cancer resection [3]. In palliative care, only about 20% of patients receive oral medication until the end of life [4], and only about 40% can be managed with a single administration route [5], resulting in a high demand for hospital preparations. Among these, suppositories have been developed as multiple hospital preparations owing to their high bioavailability, lower risk of pain, phlebitis, and bacterial infection compared to injectables, as well as their simple preparation method [6, 7].

Mianserin is classified as a tetracyclic antidepressant and exhibits relatively weaker anticholinergic effects than tricyclic antidepressants [8]. Although Anafranil is a tricyclic antidepressant approved in Japan and both oral and parenteral (intravenous) formulations are available, tetracyclic antidepressants are only available as oral formulations. Mianserin has been reported to exert additional effects beyond its primary antidepressant action, improving delirium [9]. Delirium is a psychiatric symptom that significantly impairs independence and causes severe psychological and social distress in patients and their families, making its management crucial [10]. However, in Japan, only the tablet formulation of mianserin has been approved, making it unavailable to patients with difficulty swallowing. Furthermore, in cases of terminal delirium, there are risks, such as aspiration with oral medications or self-removal of intravenous lines with injectable formulations, making suppository administration ideal. Therefore, mianserin suppositories are used for hospital preparations in clinical settings. The optimal excipient for mianserin suppositories has been previously investigated, and among the three agents, Witepsol H15, W35, and S55—Witepsol H15 have been shown to provide the highest drug release rates [11]. Regarding bioavailability in humans, a comparative study of mianserin tablets and suppositories showed that while the suppository had a higher AUC than the tablet, its Cmax was lower, indicating slower absorption compared to typical suppositories [12]. Furthermore, the authors conducted a comparative study of mianserin suppositories and tablets regarding their delirium-improving effects in clinical settings and reported that both formulations demonstrated equivalent delirium-improving effects [13].

Mianserin suppositories has been the subject of extensive basic and clinical research among hospital preparations. However, to date, no studies have investigated their long-term stability. Hospital preparations are classified based on their manufacturing processes and intended uses. Mianserin suppositories fall under Class II (When a drug approved under the Pharmaceutical Affairs Law is used for therapeutic or diagnostic purposes outside the approved scope under the Pharmaceutical Affairs Law, and the invasiveness to the human body is relatively minor). Quality control, including the long-term stability of hospital preparations, should be conducted as needed. However, no clear standards exist for testing methods or intervals, and practices vary among facilities [14]. Therefore, to ensure efficacy at the time of patient administration and to prevent waste of pharmaceuticals due to unnecessary disposal after manufacturing, we investigated the changes in content over time, sterility, and appearance of mianserin suppositories.

## Methods

### Preparation and storage of mianserin suppositories

**Fig. 1:**
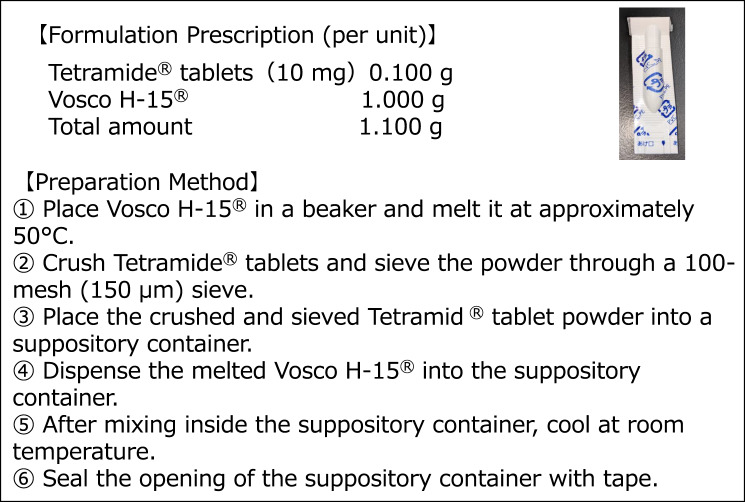
Method for preparing mianserin suppositories

The pharmaceuticals and equipment used for preparing mianserin suppositories were Tetramid^Ⓡ^ tablets 10 mg (Organon Ltd., Tokyo), Vosco H-15^Ⓡ^ (Maruishi Pharmaceutical Co., Ltd., Osaka), Suppository Container S 1.35 mL (Maruishi Pharmaceutical Co., Ltd., Osaka), and Surgical Tape-21N^Ⓡ^ (Nichiban Co., Ltd., Tokyo). Mianserin suppositories were prepared as shown in Fig. 1. Prepared mianserin suppositories were stored for 24 weeks under five conditions: RL, room temperature (15–30 °C) diffused light (1,000–1,500 lx); RD, room temperature in the dark; CL, cool temperature (2–8 °C) with diffused light; CD, cool temperature in the dark; and FD, frozen temperature (−20 °C) in the dark.

### Long-term chemical stability test

Using high-performance liquid chromatography (HPLC), the component contents were determined at 0 (immediately after preparation), 2, 4, 8, 16, and 24 weeks. Five samples were analyzed for each condition. Referencing the quantitative method for lidocaine suppositories [15], HPLC samples were prepared from each mianserin suppository using the heated dissolution and extraction method as follows. Butyl p-hydroxybenzoate (Kanto Chemical, Tokyo) was dissolved in methanol (Fujifilm Wako Pure Chemical, Osaka): 35–37% hydrochloric acid (Kanto Chemical, Tokyo) (200:1) to prepare a 100.0 mg/L internal standard solution. Place 40 mL of the internal standard solution and the mianserin suppository in a centrifuge tube. Heat and dissolve the mixture in a 50 °C water bath, stirring occasionally. After the mianserin suppository was completely dissolved, it was cooled in an ice bath for 20 min to precipitate the excipient components and centrifuged at 3,500 rpm for 5 min. Accurately measure 0.08 mL of the supernatant in an autosampler vial and add 0.92 mL of methanol: 37% hydrochloric acid (200:1) solution.

The HPLC system used for the analysis was a Nexera Lite (pump: LC-40D, detector: SPD-M40, autosampler: SIL-40C) (Shimadzu Corporation, Kyoto). The analytical column used was Wrapsil C18-L (4.6φ × 150 mm, 5 μm; JASCO Engineering, Tokyo). The column temperature was 40 °C, the flow rate was 1.5 mL/min, the injection volume was 20 μL, and the detection wavelength was 280 nm. The mobile phase was acetonitrile (Fujifilm Wako Pure Chemical, Osaka), methanol (Fujifilm Wako Pure Chemical, Osaka), and ammonium acetate (Hayashi Pure Chemical, Osaka) solution [10 mM] = 3:10:7. The detection time was set to 8 min. Standard solutions were prepared by stepwise adjustment of mianserin suppositories (0.1, 0.5, 1.0, 2.5, 5.0, 7.5, 10.0, 15.0 mg/suppository as mianserin). These standard solutions, prepared using the same heating, dissolution, and extraction methods described above, were analyzed to establish a calibration curve. The concentration range of the calibration curve was 0.2 to 30.0 μg/mL (0.2, 1.0, 2.0, 5.0, 10.0, 15.0, 20.0, 30.0 μg/mL). The calibration curve, prepared using the internal standard method described previously, had an R2 value of 0.999 or higher. The detection limit (S/N ratio of 3) calculated from the S/N ratio was 0.2 μg/mL, and the quantification limit (S/N ratio of 10) was 0.6 μg/mL. The acceptable content specification for the clinical use of hospital preparations was defined as 90–110% [16].

Data are expressed as mean ± standard error (SE). For the comparison of storage conditions among the five groups at 24 weeks, Tukey–Kramer tests were performed with a significance level of 0.05, using the Bell Curve for Excel ver 0.4.08 (Social Survey Research Information Co., Ltd., Tokyo).

### Microbial limit test

Microbial testing of nonsterile products listed in the Japanese Pharmacopoeia was conducted according to the test method for lipid products applicable to this suppository. Colony-forming units (CFU) per gram of suppository were measured using the agar dilution method after preparation, after 24 weeks of storage at room temperature under diffuse light, and 24 weeks of storage in the dark (*n* = 4). For the total aerobic microbial culture, Soybean-Casein Digest agar with Lecithin & Polysorbate 80 (SCDLP) medium (Shioya MS, Hyogo) was used, and the cultures were incubated at 35 °C for 3 days. For aerobic fungal cultures, Sabouraud-Dextrose Agar with Lecithin & Polysorbate 80 (SALP) medium (Shioya MS, Hyogo) was used for 7 days at 27 °C. Data are expressed as mean ± standard deviation (SD). The acceptable detection limits for bacterial and fungal counts in clinical use were established as ≤ 1000 CFU/1 g and ≤100 CFU/1 g, respectively, due to the rectal route of administration [17].

### Appearance change test

Under diffused light (1,500 lux), a single investigator visually inspected the suppositories under each condition at 0, 2, 4, 8, 16, and 24 weeks to determine whether any color changes were present.

## Results

### Long-term chemical stability test

**Fig. 2:**
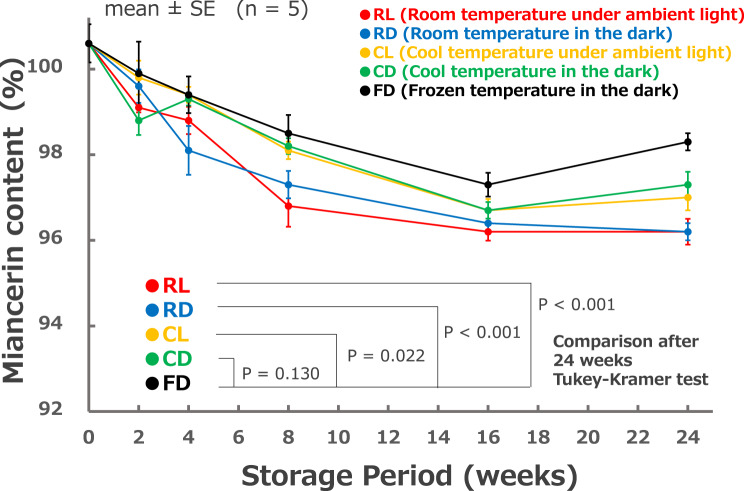
Long-term chemical stability test

As shown in Fig. 2, a slight decrease was observed over time under all storage conditions. However, the content after 24 weeks (mean ± SE, *n* = 5) was as follows: RL, 96.2 ± 0.3%; RD, 96.2 ± 0.2%; CL, 97.0 ± 0.3%; CD, 97.3 ± 0.3%; and FD, 98.3 ± 0.2%. These values meet the acceptable content specifications for clinical use in hospital preparations. Comparisons between groups revealed significant differences in FD content at 24 weeks compared with RL, RD, and CL (*p* < 0.05). No significant differences were observed between the FD and CD groups and between any of the other groups.

### Microbial limit test

As shown in Table 1, the samples met the criteria for total aerobic microorganisms ≤1000 CFU/1 g and total fungi ≤100 CFU/1 g under all conditions, immediately after preparation and after 24 weeks of storage in RL and FD.

**Table 1:** Microbial limit test

CFU/1 g of Suppository (Mean ± SD)	SALP (*n* = 4)	SCDLP (*n* = 4)
Immediately after preparation	3.75 ± 2.5^†^	0 ± 0^‡^
After 24 weeks at room temperature (15–30°C) under ambient light	0 ± 0^†^	0 ± 0^‡^
After 24 weeks at frozen temperature (−20°C) in the dark	0 ± 0^†^	0 ± 0^‡^

† Reference Values in the Japanese Pharmacopoeia, 18th Edition < 100 CFU

‡ Reference Values in the Japanese Pharmacopoeia, 18th Edition < 1,000 CFU

CFU: Colony-forming unit, SALP: Sabouraud-dextrose agar with lecithin & polysorbate 80, SCDLP: Soybean-casein digest agar with lecithin & polysorbate 80, SD: Standard Deviation.

### Appearance change test

**Fig. 3:**
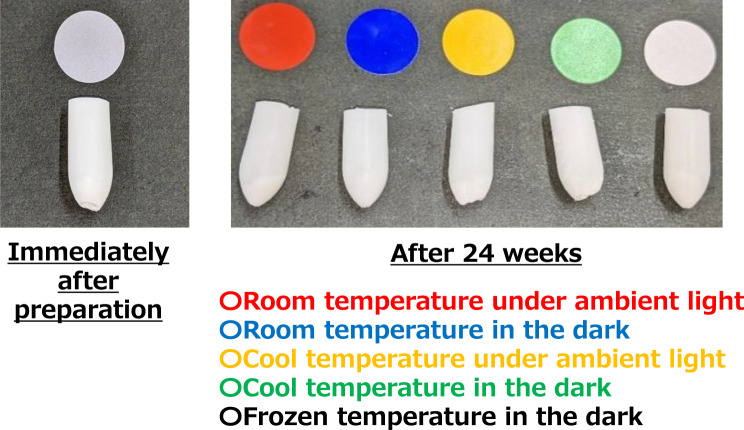
Appearance change test

As shown in Fig. 3, no crystallization or discoloration was observed in the suppositories from 0 (immediately after preparation) to 24 weeks under all conditions; the suppositories remained white.

## Discussion

The results of this study demonstrated that mianserin suppositories maintained a content level above 95% for six months after preparation under all storage conditions, ranging from RL to frozen FD, with no observable changes in appearance. Furthermore, microbial limit tests showed that after 24 weeks of storage in RL or FD, the levels for bacterial and fungal were significantly below the acceptable standards (as ≤ 1000 CFU/1 g and ≤100 CFU/1 g). In contrast, significant differences were observed between RL, RD, and CL compared with FD. Therefore, storage in FD or a CD is more desirable. Storage at the FD depends on the availability of equipment at the affiliated facility. Furthermore, during hot summer days, when temperatures exceed 30 °C, the base agent may melt, making room-temperature storage unsuitable for quality maintenance. Therefore, basic storage on the CD is considered optimal. Additionally, during temporary movements such as overnight stays outside the hospital, room-temperature storage is considered unlikely to cause significant problems.

The chemical structure of mianserin consists of a robust four-ring skeleton containing aromatic rings. The tablets remain unchanged for 36 months when stored at room temperature in open glass bottles and remain unchanged for 4 months even at 40 °C and 75% humidity [18]. Furthermore, since mianserin does not possess functional groups that react with the base compound, witepsol (glycerin + fatty acids), it is considered to exhibit high long-term stability even after mixing with the base compound. Regarding microbial limit tests, aerobic fungi were detected immediately after preparation. However, during storage fungi die because of nutrient depletion including water and nitrogen sources, within the witepsol [19]. In clinical settings, the microbial load immediately after preparation depends on the quality of the sterile equipment used to suppress airborne bacteria and the skill of the preparer. Therefore, pharmacists responsible for preparing hospital preparations must exercise extreme caution [20].

In addition to mianserin suppositories, haloperidol injection is available as a treatment option for delirium patients who have difficulty taking oral medications [10]. However, haloperidol is contraindicated in patients with Parkinson’s disease or dementia with Lewy bodies due to its D_2_ antagonist effects. A previous report indicates that mianserin has delirium-improving effects comparable to haloperidol [21], and there is a clinically significant need for mianserin suppositories that can be administered to these patients. This study demonstrates the long-term stability of mianserin suppositories and can be considered beneficial for the development from hospital preparation to approved pharmaceutical. On the other hand, the maximum duration of the long-term evaluation in this study was 24 weeks, and it was unclear at what point stability would no longer be maintained. As a future direction, we plan to conduct long-term evaluations lasting one year (52 weeks) or longer.

Hospital preparations allow the use of new dosage forms and dosages tailored to individual patients; however, compared with approved pharmaceuticals, they have limited stability and accumulated clinical data. In 2012, the Japanese Society of Hospital Pharmacists established the “Guidelines for the Making and Use of Hospital Preparations” 14); however, it did not specify clear standards for quality testing for each preparation. Furthermore, due to limitations in the analytical equipment available at individual medical institutions and the use of empirical storage methods, it has been reported that only 3% of hospitals actually perform quality testing [22]. This study demonstrates that the quantitative method for lidocaine suppositories can also be applied to mianserin suppositories. In recent years, olanzapine suppositories [6] and quetiapine suppositories [23] have been developed as hospital preparations for patients with delirium who have difficulty taking oral medications, similar to mianserin suppositories. While these reports demonstrate an improvement in delirium following administration of the hospital preparations, long-term stability test of the ingredients has not been conducted. Therefore, resolving the clinical question of uncertainty regarding how long the hospital preparation can be stored represents one of the unique strengths of this research. Since these also use Vosco H-15^Ⓡ^ as the base agent, it is anticipated that applying the same methodology as this study will enable investigation of their long-term chemical and biological stability. In future research, it will be important to provide evidence-based medicine rather than relying on empirical storage methods, and to substantiate the shelf life of hospital preparations.

## Conclusion

The results of testing the long-term chemical and biological stability in this study showed that the mianserin suppository maintained good component retention above 95% from immediately after preparation to 24 weeks later under all storage conditions, ranging from RL to FD.

## Data Availability

All data is provided.

## Abbreviations

CFU: Colony-forming units
CD: Cool temperature in the dark
CL: Cool temperature under ambient light
FD: Frozen temperature in the dark
HPLC: High-performance liquid chromatography
RD: Room temperature in the dark
RL: Room temperature under ambient light
SALP: Sabouraud-dextrose agar with lecithin & polysorbate 80
SCDLP: Soybean-casein digest agar with lecithin & polysorbate 80
SD: Standard deviation
SE: Standard error

## References

[1] Hanawa T. In-hospital preparations for the assistance of the medication evidence based quality management of the In-hospital formulations. J Pharm Sci Technol Jpn. 2012;72:9–14.

[2] Eslick GD, Talley NJ. Dysphagia: epidemiology, risk factors and impact on quality of life―a population-based study. Aliment Pharmacol Ther. 2008;27:971–79. 10.1111/j.1365-2036.2008.03664.x 18315591

[3] Frowen J, Hughes R, Skeat J. The prevalence of patient-reported dysphagia and oral complications in cancer patients. Support Care Cancer. 2020;28:1141–50. 10.1007/s00520-019-04921-y 31203510

[4] Masman AD, van Dijk M, Tibboel D, Baar FP, Mathôt RA. Medication use during end-of-life care in a palliative care centre. Int J Clin Pharm. 2015;37:767–75. 10.1007/s11096-015-0094-3 PMC4594093 25854310

[5] Coyle N, Adelhardt J, Foley KM, Portenoy RK. Character of terminal illness in the advanced cancer patient: pain and other symptoms during the last four weeks of life. J Pain Symptom Manage. 1990;5:83–93. 10.1016/s0885-3924(05)80021-1 2348092

[6] Matsumoto K, Kimura S, Takahashi K, Yokoyama Y, Miyazawa M, Kushibiki S, et al. Pharmaceutical studies on and clinical application of olanzapine suppositories prepared as a hospital preparation. J Pharm Health Care Sci. 2016;2:20. 10.1186/s40780-016-0055-6 PMC5030735 27672443

[7] Sato J, Fujimoto T, Umeda S, Tsukagoshi M, Tanaka R. Experience with preparation and use of hospital preparation of perospirone suppositories for patients with delirium. Jpn J Pharm Palliat Care Sci. 2023;16:47–51.

[8] Nakamura J, Uchimura N. Mianserin in treatment of delirium and its mechanism. J Clin Exp Med. 1995;13:1024–25.

[9] Nakamura J, Uchimura N, Yamada S, Nakazawa Y. Does plasma free-3-methoxy-4-hydroxyphenyl(ethylene)glycol increase in the delirious state? A comparison of the effects of mianserin and haloperidol on delirium. Int Clin Psychopharmacol. 1997;12:147–52. 9248871 10.1097/00004850-199705000-00005

[10] Japan Psycho-Oncology Society. Japanese association of supportive care in cancer. Clinical guidelines for delirium in adult cancer patients (2025 version). Tokyo: Kanehara & Co., Ltd; 2025.

[11] Nakajima T, Iwata M, Nawata S, Saito H, Nakamura Y, Kobayashi Y, et al. Physicopharmaceutical approach for hospital preparation of mianserin hydrochloride suppositories. Jpn J Pharm Health Care Sci. 2012;38:702–07.

[12] Nawata S, Kohyama N, Uchida N, Numazawa S, Ohbayashi M, Kobayashi Y, et al. The pharmacokinetics of mianserin suppositories for rectal administration in dogs and healthy volunteers: a pilot study. J Pharm Health Care Sci. 2016;2:12. 27190632 10.1186/s40780-016-0046-7 PMC4869351

[13] Tanaka R, Ishikawa H, Sato T, Shino M, Matsumoto T, Omae K, et al. Delirium improvement with mianserin suppositories in cancer patients. Clin Oncol (Belmont). 2016;1:1127.

[14] The Japanese Society of Hospital Pharmacists. Guidelines for the making and use of hospital preparations. https://www.jshp.or.jp/activity/guideline/20230206-2.pdf. revised in 2023 January. 2023. Accessed 22 Dec 2025.

[15] Kase N, Yazaki H, Fukushima E. Determination of lidocaine in suppositories. Bull of The Public Health Lab of Chiba Prefecture. 1996;20:18–22.

[16] Sakakibara T, Kume T, Harada S, Ikeuchi S, Morimoto A, Shino M. Stability of mouthwash based on betamethasone sodium phosphate. J Jpn Soc Hosp Pharm. 2022;58:173–76.

[17] The Japanese ministry of Health, labour and welfare. The Jpn Pharmacopoeia, 18th Edition. 2021. https://www.pmda.go.jp/rs-std-jp/standards-development/jp/0192.html. Accessed 22 Dec 2025.

[18] Organon-Japan. Tetramide® tablets interview form. 17th ed. Revised in Feb 2024.

[19] Peleg M. A New look at models of the combined effect of temperature, pH, water activity, or other factors on microbial growth rate. Food Eng Rev. 2022;14:31–44.

[20] Yanagihara Y. Role and task of Pharmaceutical manufacturing in clinical practice. J Pharm Sci Technol Jpn. 2012;72:20–25.

[21] Nakamura J, Uchimura N, Yamada S, Nakazawa Y. Effects of mianserin hydrochloride on delirium: comparison with the effects of oxypertine and haloperidol. Jpn J Neuropsychopharmacol. 1994;14:269–77. 7975931

[22] Kishimoto K, Nagashima M, Shigeoka S, Minowa K, Kadoi H, Moriyasu T, et al. Quality control for hospital preparation of medicine - the stability test -. Ann Rep Tokyo Metr Inst Pub Health. 2006;57:93–99.

[23] Takeuchi K, Koh M, Tamura A, Amasaki M, Ueda H. Experience in using In-hospital formulation quetiapine suppositories for delirium in cancer patients. Palliat Care Res. 2017;12:717–22.

